# Extraventricular Neurocytoma of the Sellar Region Presenting With Syndrome of Inappropriate Antidiuresis

**DOI:** 10.1210/jcemcr/luae099

**Published:** 2024-08-16

**Authors:** Elisa Lamback, Ferdinand Duenas Cabrera Filho, Nina Ventura, Leila Chimelli, Mirjam Christ-Crain, Mônica R Gadelha

**Affiliations:** Neuroendocrinology Research Center, Endocrinology Section, Medical School and Hospital Universitário Clementino Fraga Filho, Universidade Federal do Rio de Janeiro, Rio de Janeiro 21941-617, Brazil; Neuropathology and Molecular Genetics Laboratory, Instituto Estadual do Cérebro Paulo Niemeyer, Secretaria Estadual de Saúde, Rio de Janeiro 20231-092, Brazil; Neuroendocrine Unit, Instituto Estadual do Cérebro Paulo Niemeyer, Secretaria Estadual de Saúde, Rio de Janeiro 20231-092, Brazil; Neuroradiology Unit, Instituto Estadual do Cérebro Paulo Niemeyer, Secretaria Estadual de Saúde, Rio de Janeiro 20231-092, Brazil; Neuroradiology Unit, Instituto Estadual do Cérebro Paulo Niemeyer, Secretaria Estadual de Saúde, Rio de Janeiro 20231-092, Brazil; Neuropathology and Molecular Genetics Laboratory, Instituto Estadual do Cérebro Paulo Niemeyer, Secretaria Estadual de Saúde, Rio de Janeiro 20231-092, Brazil; Department of Endocrinology, Diabetology and Metabolism, University Hospital Basel, Basel 4001, Switzerland; Department of Clinical Research, University Hospital Basel, University of Basel, Basel 4001, Switzerland; Neuroendocrinology Research Center, Endocrinology Section, Medical School and Hospital Universitário Clementino Fraga Filho, Universidade Federal do Rio de Janeiro, Rio de Janeiro 21941-617, Brazil; Neuropathology and Molecular Genetics Laboratory, Instituto Estadual do Cérebro Paulo Niemeyer, Secretaria Estadual de Saúde, Rio de Janeiro 20231-092, Brazil; Neuroendocrine Unit, Instituto Estadual do Cérebro Paulo Niemeyer, Secretaria Estadual de Saúde, Rio de Janeiro 20231-092, Brazil

**Keywords:** extraventricular neurocytoma, syndrome of inappropriate antidiuresis, arginine vasopressin, sellar mass

## Abstract

Neurocytomas are neuronal tumors that are usually intraventricular. Rare cases can arise from extraventricular sites. To our knowledge, only 29 cases of extraventricular neurocytoma of the sellar region (EVNSR) have been reported in the literature. We describe a case of a 39-year-old woman who presented with a one-month history of refractory headache, nausea and vomiting. Magnetic resonance imaging (MRI) showed a 5.1 × 3.1 × 2.2 cm sellar and suprasellar mass, suggestive of a pituitary adenoma (PA). She had hyponatremia, obstructive hydrocephalus, and panhypopituitarism at presentation (hypogonadism, adrenal insufficiency). After glucocorticoid replacement therapy and ventriculoperitoneal shunt, the vomiting and headache resolved, but she remained with nausea and hyponatremia. She was submitted to surgery, and histopathological analysis revealed a neurocytoma with positive immunostaining for arginine vasopressin. Syndrome of inappropriate antidiuresis (SIAD) was diagnosed but did not resolve after surgery due to residual tumor, despite fluid restriction and saline replacement. SIAD later resolved with empagliflozin. In conclusion, EVNSR is extremely rare and can be misdiagnosed as PA on MRI. In the context of SIAD and extraventricular neurocytoma, a secreting arginine vasopressin tumor must be considered. SIAD can be challenging to treat, with excision of the EVNSR the treatment choice and, alternatively, empagliflozin associated with fluid restriction.

## Introduction

Neurocytomas are neuronal tumors that are usually intraventricular (1). In rare occasions, they arise from extraventricular sites (1). Extraventricular neurocytomas have an annual incidence of 0.01 cases per 100 000 individuals and a peak incidence in the third and fourth decades (1). The most frequently documented extraventricular locations are the cerebral hemispheres, cerebellum, spinal cord, thalamus, and pons (1). Exceedingly rare are neurocytomas arising in cranial nerves, cauda equina, and sellar region (1). To our knowledge, only 29 cases of extraventricular neurocytoma of the sellar region (EVNSR) have been reported in the literature (2-19). We present a case of EVNSR and review the literature.

## Case Presentation

**Figure 1.**
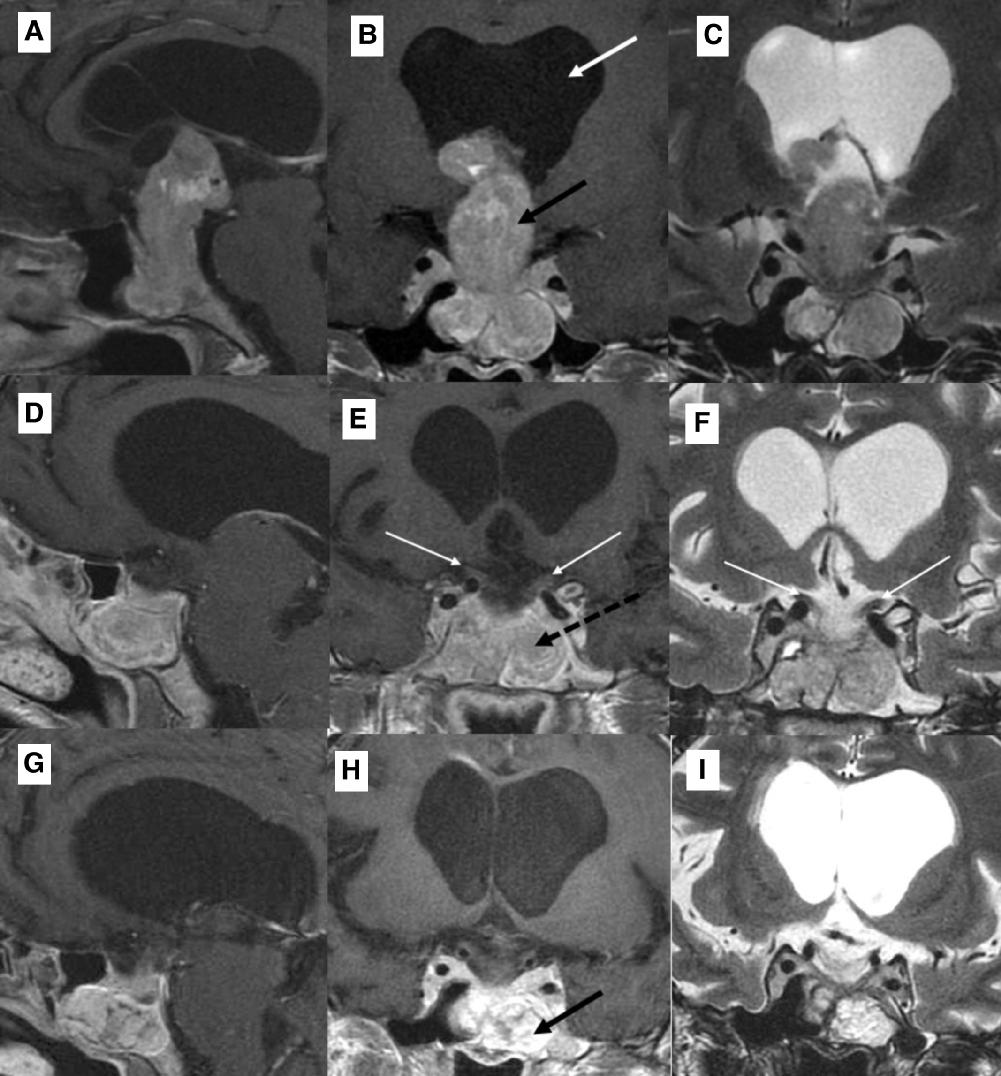
Sellar MRI. Sagittal T1 post-contrast (A, D, G), coronal T1 post-contrast (B, E, H), coronal T2 (C, F, I) views. (A-C) Preoperative MRI showing a 5.1 × 3.1 × 2.2 cm sellar, supra-, infra-, and para-sellar mass (black arrow) that occupied the sphenoid sinuses inferiorly and invaded both cavernous sinus. The lesion had significant vertical growth, invaded the third ventricle, and caused obstructive hydrocephalus (white arrow). The lesion displayed heterogeneous contrast enhancement, was slightly hyperintense on T1 and T2, and was suggestive of a pituitary macroadenoma. The optic chiasm, pituitary gland, and stalk were not individualized. (D-F) Three months after the first surgery, residual mass could be seen in the sphenoid sinus (dashed black line) with the optic chiasm decompressed and pulled down (white arrows). (G-I) One month after the second surgery, residual lesions in both cavernous sinuses could be seen, as well as fat graft that was inserted during surgery for hemostasis (black arrow). Abbreviations: MRI, magnetic resonance imaging.

We report a case of a 39-year-old woman who presented with a 1-month history of refractory holocranial headache, nausea, and vomiting. Magnetic resonance imaging (MRI) showed a 5.1 × 3.1 × 2.2 cm (longitudinal × transverse × antero-posterior diameters) invasive sellar, infra-, and suprasellar lesion, causing obstructive hydrocephalus, suggestive of a pituitary adenoma (PA) (Fig. 1A-1C). On physical examination, blood pressure was 134 × 90 mmHg, and the patient did not have visual field loss or motor deficits. She had hypogonadism (amenorrhea for the last 4 months) and adrenal insufficiency [morning serum cortisol 1.4 mcg/dL (38.6 nmol/L) (low when < 3 mcg/dL; < 83 nmol/L)]. TSH was 0.3 mIU/L (reference 0.4-5.8 mIU/L), free T4 1.1 ng/dL (142 nmol/L) (reference 0.7-1.8 ng/dL; 90-232 nmol/L), diluted prolactin 7.1 mcg/L (reference 4.8-23.3 mcg/L), and IGF-I 117 ng/mL (15.3 nmol/L) (reference 63-223 ng/mL; 8-29 nmol/L). At baseline, sodium concentrations were low (121-123 mEq/L) (reference 135-145 mEq/L). She was previously well and did not take any prior medication. No hyporexia, dizziness, fainting, weight loss, or abdominal pain was reported. After glucocorticoid replacement therapy and ventriculoperitoneal shunt, the headache and vomiting resolved but not the nausea or hyponatremia.

## Diagnostic Assessment

**Figure 2.**
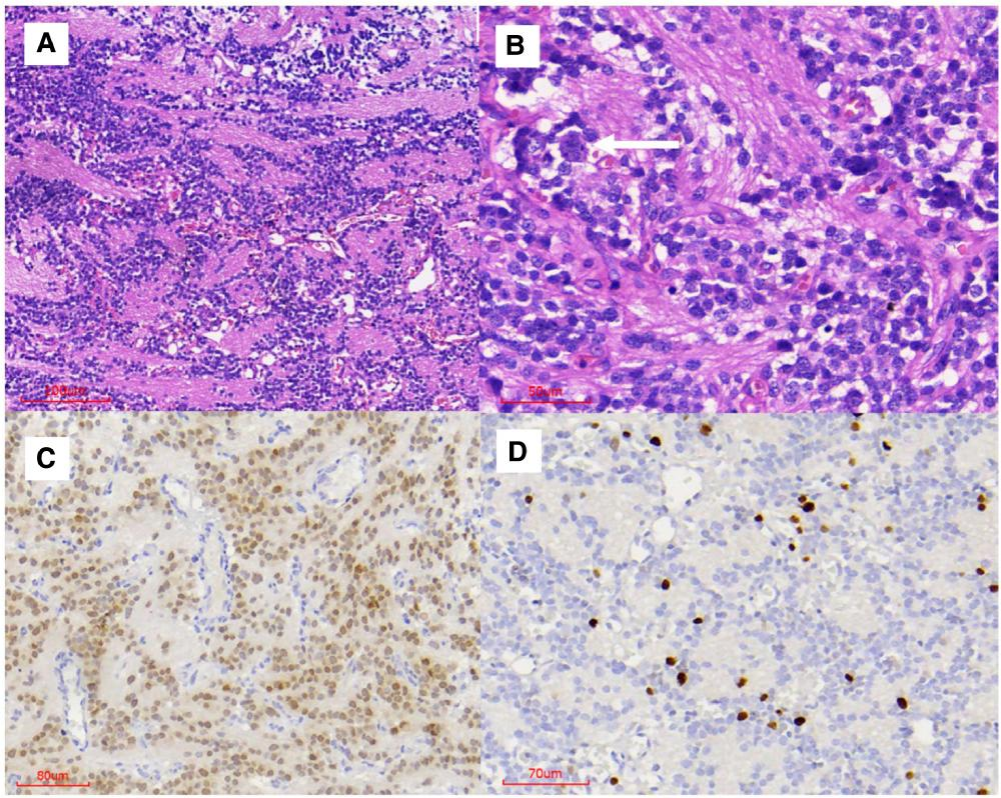
Histological analysis. **(**A and B) Hematoxylin and eosin exhibiting a monomorphic neoplasia formed by rows of cell clusters, separated by neuropil islands. An occasional large cell with eosinophilic cytoplasm can be observed in B (arrow). (C) Positive neuronal nuclear antigen immunostaining. (D) Ki-67 of 10% in a hotspot.

**Figure 3.**
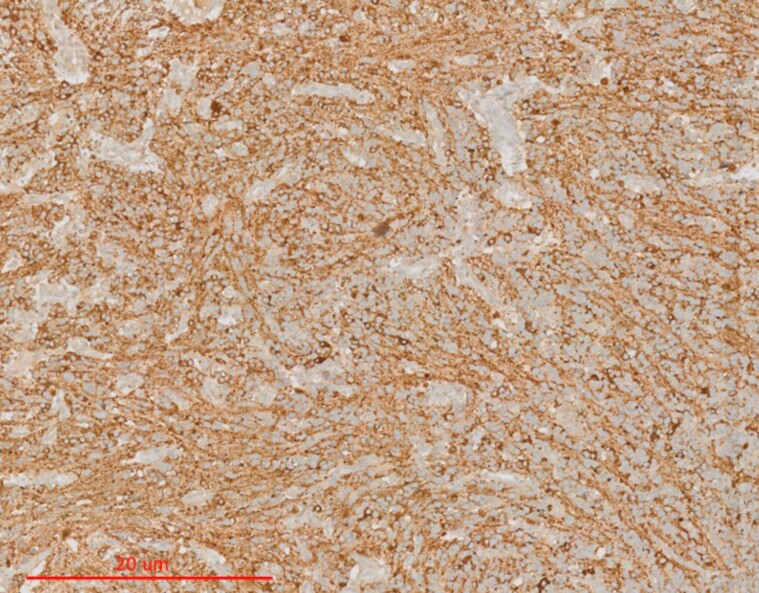
Anti-arginine vasopressin immunostaining. Positive anti-arginine vasopressin (dilution 1:400).

Syndrome of inappropriate antidiuresis (SIAD) was diagnosed based on serum osmolality of 266 mOsm/kg (below 275 mOsm/kg), urinary sodium excretion of 48 mEq/L (above 30 mEq/L), and inappropriate urine concentration of 428 mOsmol/kg (above 100 mOsmol/kg), associated with clinical euvolemia (20). The patient was submitted to surgery, and the diagnosis was made by histological analysis based on the morphological appearances on hematoxylin and eosin analysis and the immunohistochemical results (diffuse cytoplasmatic immunoreactivity for synaptophysin and positive neuronal nuclear antigen) (Fig. 2). The neoplasia was formed by relatively monomorphic cells with small uniform nuclei and scarce or clear cytoplasm, arranged in sheets, rows, or cell clusters with neuropil islands. Occasional larger cells with eosinophilic cytoplasm were also observed. Hyalinized vessels were present. One mitosis was seen in 10 high-power fields. No necrosis or microcalcifications were seen. Ki-67 was 10% in hotspots (Fig. 2). The vast majority of the tumor cells were positive for anti-arginine vasopressin antibody (self-made polyclonal rabbit antibody) (21) (Fig. 3).

## Treatment

**Figure 4.**
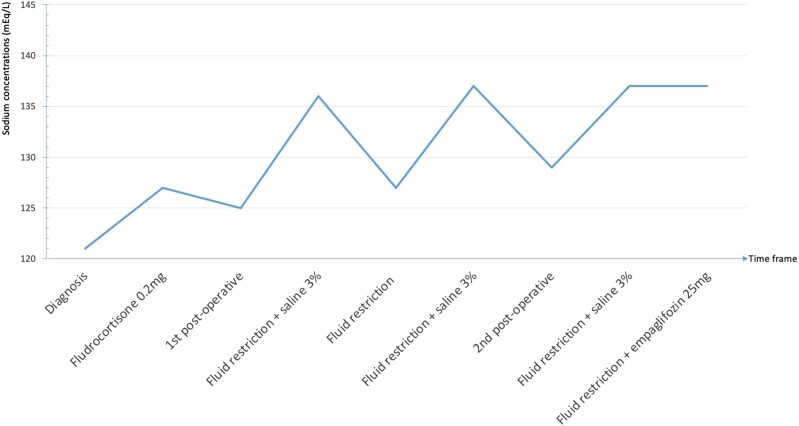
Sodium concentrations over time.

The patient was submitted to transcranial surgery. After surgery, she developed central hypothyroidism that was adequately replaced. SIAD did not resolve, despite fluid restriction of 1L/day during the period she was hospitalized. Without saline (3%) replacement, sodium concentrations fell below 130 mEq/L, and the patient developed symptoms of nausea, vomiting, and hyporexia. MRI showed a residual sellar lesion (Fig. 1D-1F), and she was submitted to a transsphenoidal surgery 3 months after the first surgery but remained hyponatremic due to the presence of residual lesions in both cavernous sinuses (Fig. 1G-1I). SIAD later resolved with empagliflozin 25 mg/day and fluid restriction of 1L/day, with sodium concentrations varying from 134 to 137 mEq/L (Fig. 4). Glucose concentrations remained normal during the 1-month use of empagliflozin [77-95 mg/dL (4.3-5.2 mmol/L), reference 70-100 mg/dL (3.9-5.5 mmol/L)].

## Outcome and Follow-up

Unfortunately, the patient developed multiple surgical and clinical complications (lacunar infarction, intracranial hemorrhage, 2 reversed cardiac arrests, venous thrombosis, multiple sepsis, blood dyscrasia) and died after 5 months of hospitalization.

## Discussion

To our knowledge, only 29 cases of EVNSR have been described to date (Table 1). The etiology of EVNSR is unknown, with no risk factor or inherited genetic susceptibility described (1). However, based on a literature review, the case reports originated mostly from Asian countries (21 of 29 cases), suggesting that there might be an ethnical predilection with Chinese patients more affected (17 cases). The majority were females (18 cases) and presented at a median age of 40 years old (ranging from 4 to 70 years old) similar to our case (Table 1).

**Table 1.** Literature review of previous cases

Case no	Publication	Origin	Sex	Age (years)	Signs and symptoms	Preoperative pituitary function	SIAD	Anti-AVP	Location	Imaging	Outcome	Ki-67(%)	Prognosis (follow-up period)
1	Maguire et al, 1992 (2)	Canada	F	55	Visual	Normal	N/A	Positive in most tumor cells (Sera Lab)	Sellar and SS	Suggestive of PA Not-invasive	GTR Transient AVP deficiency	N/A	No recurrence
2	Yang et al, 2009 (3)	China	F	46	Visual	N/A	N/A	N/A	Sellar and SS	Presence of calcifications Invasive Suggestive of meningioma	STR	N/A	N/A
3	Pang et al, 2010 (25)	China	F	66	Visual, headache	N/A	N/A	N/A		N/A	STR	N/A	N/A
4	Wang et al, 2012 (5)	UK	F	50	Visual	Normal	N/A	N/A	Sellar and SS	Speckled calcifications (CT) Invasive Suggestive of PA or craniopharyngioma	STR, RT	N/A	No recurrence (18 months)
5	Zhu et al, 2012 (26)	China	F	32	Visual	N/A	N/A	N/A	N/A	Suggestive of craniopharyngioma	STR, RT	N/A	N/A
6	Liu et al, 2013 (6)	China	M	40	Visual	N/A	N/A	N/A	Sellar and SS	Small foci of cystic degeneration Invasive Suggestive of PA	N/A	N/A	N/A
7	Wang et al, 2013 (7)	China	F	23	Visual, headache	Normal	N/A	N/A	Sellar and SS	Invasive Suggestive of PA	STR, RT	N/A	N/A
8	Kawaji et al, 2014 (8)	Japan	M	48	Visual	N/A	N/A	N/A	Sellar and SS	Suggestive of PA	STR, RT	N/A	Cranial dissemination (6 years)
9	Xiong et al, 2015 (9)	China	N/A	56	N/A	N/A	N/A	N/A	Sellar	N/A	N/A	N/A	N/A
10	Makis et al, 2015 (10) Asa et al, 2019 (17)	Canada	F	34	Amenorrhea, hyponatremia	Hypogonadism	Present	Diffuse and strong cytoplasmatic reactivity (Peninsula)	Sellar and SS	Invasive Suggestive of PA	STR, RT, lutetium Fluid restriction	3-8	Multiple recurrences (during 26 years)
11	Peng et al, 2015 (11)	China	M	56	Headache	Normal	N/A	N/A	Sellar and SS	Invasive Suggestive of PA	GTR	0-1	N/A
12	Chen et al, 2016 (27)	China	F	50	Visual	Normal	N/A	N/A	Sellar and SS	Suggestive of PA	STR, RT	2	No recurrence (36 months)
13	Chen et al, 2016 (27)	China	M	62	Visual	Hypogonadism	N/A	N/A	Sellar and SS	Invasive Suggestive of PA	STR, RT	<2	No recurrence (36 months)
14	Cho et al, 2016 (12)	South Korea	M	14	Visual	N/A	N/A	N/A	Sellar and SS	Invasive Glioma	STR, RT	<1	Recurrence (12 months)
15	Wang et al, 2016 (13)	China	M	25	Visual	Normal	N/A	N/A	Sellar and SS	Calcification (CT) Invasive Suggestive of PA or meningioma	STR, transient AVP deficiency	3	N/A
16	Xu et al, 2017 (14)	China	M	23	Headache	N/A	N/A	N/A	Sellar	N/A	GTR	0-8	No recurrence (59 months)
17	Xu et al, 2017 (14)	China	F	4	Headache, vomiting	N/A	N/A	N/A	Sellar	N/A	STR, RT		Recurrence (49 months) Death
18	Xu et al, 2017 (14)	China	F	58	Visual, headache	N/A	N/A	N/A	Sellar	N/A	STR, RT		No recurrence (45 months)
19	Tan et al, 2019 (4)	Singapore	F	59	Visual, nausea, vomiting	Normal	Present	N/A	Sellar and SS	Invasive Suggestive of PA	STR	<1	N/A
20	Nery et al, 2019 (15)	Brazil	M	27	Visual	Panhypopituitarism	N/A	N/A	Sellar and SS	Non-invasive Suggestive of PA	GTR, AVP deficiency	0.8	No recurrence (18 months)
21	Tish et al, 2019 (16)	USA	M	70	Imbalance, dizziness	N/A	N/A	N/A	SS	N/A	STR, RT	6-7	No recurrence (12 months)
22	Asa et al, 2019 (17)	Canada	F	39	Hyponatremia	Normal	Present	Diffuse and strong cytoplasmatic reactivity (Peninsula)	Sellar and SS	Invasive Suggestive of PA	STR Fluid restriction, furosemide	4-12	N/A
23	Asa et al, 2019 (17) Zhang et al, 2019 (18)	Canada	M	17	Nausea, vomiting, dysarthria, hyponatremia	Panhypopituitarism	Present		Sellar and SS	Invasive Calcification Suggestive of PA	STR, tolvaptan, fludrocortisone, Lanreotide		Stable residual mass (48 months)
24	Asa et al, 2019 (17)	Canada	F	40	Visual, headache	N/A	N/A		Sellar and SS	Suggestive of PA	STR		N/A
25	Karkra et al, 2022 (28)	India	F	45	Headache, vomiting	N/A	N/A	N/A	SS	Invasive Cystic areas (calcification? hemorrhage?)	STR	8	Death
26	Zhang et al, 2022 (19)	China	F	28	Visual	Normal	Normal serum vasopressin*^a^*	Positive	Sellar and SS	Invasive Suggestive of PA	STR	1-2	Recurrence (50 months)
27	Zhang et al, 2022 (19)	China	F	27	Visual	Normal	N/A	Positive	Sellar and SS	Suggestive of PA	GTR	1-2	Recurrence (118 months)
28	Zhang et al, 2022 (19)	China	F	40	Visual	Normal	N/A	Positive	Sellar and SS	Suggestive of PA	STR	1-2	No recurrence
29	Zhang et al, 2022 (19)	China	M	46	Visual	N/A	N/A	Positive	Sellar and SS	Suggestive of PA	STR	6	Recurrence (11 months)
30	Present case	Brazil	F	39	Headache nausea, vomiting, hyponatremia	Panhypopituitarism	Present	Positive in most tumor cells (in-house antibody)	Sellar and SS	Invasive Suggestive of PA	STR, fluid restriction, empaglifozin	10	Death (5 months)

Abbreviations: AVP, arginine vasopressin; CT, computed tomography; F, female; GTR, gross total resection; M, male; MRI, magnetic resonance imaging; N/A, not available; PA, pituitary adenoma; RT, radiotherapy; SIAD, syndrome of inappropriate antidiuresis; SS, suprasellar; STR, subtotal resection.

^*a*^Normal serum vasopressin, without information regarding sodium concentrations. Reviews suggest not measuring vasopressin as it can be inappropriately high in SIAD or can be degraded ex vivo (20).

The majority of patients had compressive-related symptoms such as vision loss and headache. Few had symptoms of intracranial hypertension caused by obstructive hydrocephalus, as seen in our case. Eleven patients had normal pituitary function, and 5 had at least 1 pituitary deficiency (2 had hypogonadism and 3 had panhypopituitarism) (Table 1).

Commonly, the lesions resembled PA (20 of 23 cases). In other cases, it resembled a craniopharyngioma, meningioma, or glioma. None of the cases were preoperatively diagnosed as neurocytoma.

More than half of the lesions also had heterogeneous contrast enhancement and were invasive tumors, as seen in our case. One case particularly resembled our own because of the lesion's vertical growth (6).

Sparse and small calcifications appeared in some lesions on imaging studies, which are uncommon in PA. Unfortunately, computed tomography or susceptibility-weighted imaging on MRI (that would be helpful to identify calcifications) were not available in the majority of case reports, including our own. These calcifications probably reflect microcalcifications seen in histopathology, present in some cases (1).

In pathology, extraventricular neurocytomas are diffusely positive for synaptophysin and exhibit variable neuronal nuclear antigen expression, neuronal differentiation markers (1). Some data suggest that these tumors derive from the hypothalamus and can secrete hormones such as vasopressin leading to SIAD (2, 17, 19). In only 3 studies, antivasopressin staining was performed and was described as positive, positive in most cells, or diffuse with strong cytoplasmatic reactivity (2, 17, 19). In our case, it was positive in most cells. Since the antibody does not have a positive control and some tumor cells were negative, we omitted the primary antibody to see if it would stain. There was no tumoral staining with the secondary antibody, and this was used as a negative control. Lack of a positive control is a limitation; therefore, a definite proof of arginine vasopressin production cannot be made.

Regarding treatment, all patients were submitted to surgery and, in the minority of cases (5/29 cases; Table 1), gross total resection was possible, probably because most lesions invaded the cavernous sinus. In half of the cases that presented subtotal resection (12/24 cases), patients were also treated with radiotherapy. Two patients died during follow-up. Long-term follow-up data was available in 9 patients. Five (55%) cases appeared to be radiosensitive and did not exhibit tumor recurrence after follow-up periods of 12, 18, 36, 36, and 45 months. Two (22%) cases presented with tumor recurrence after 12 and 49 months, 1 (11%) had multiple recurrences over a 26-year follow-up, and another 1 (11%) had cranial dissemination after 6 years of the initial surgery. Although extraventricular neurocytomas are described as grade 2 with Ki-67 usually low (1-3%), some cases, including ours, had a high proliferative index suggesting a more aggressive clinical behavior (1).

Unfortunately, the minority of studies described if electrolyte imbalance was present (Table 1). In these cases, hyponatremia was treated with fluid restriction, furosemide, fludrocortisone, tolvaptan, lutetium, or lanreotide as some tumors express the somatostatin receptor (17, 18). It is important to mention that the most common cause of hyponatremia in a patient with a sellar/suprasellar tumor is hypopituitarism (adrenal insufficiency) (20). Only after exclusion of these deficiencies or after their correct replacement should we consider SIAD. It is also important to bear in mind that the correction of hyponatremia has to be done gradually (< 10 mEq/day) to avoid osmotic demyelination syndrome (22).

In a recently published review about SIAD, empagliflozin was proposed as second-line treatment in patients refractory to fluid restriction (20), and this was our option since tolvaptan and urea, also second-line options, are not readily available in our country. Sodium-glucose cotransporter 2 inhibitors act in the proximal tubule to reduce glucose and sodium reabsorption leading to glycosuria and increased water excretion due to osmotic diuresis. In 2 clinical trials, sodium concentrations raised 3 and 10 mEq/L with empagliflozin 25 mg/day alone and associated with fluid restriction, respectively (23, 24). Empaglifozin effectively normalized sodium concentrations in our patient, but it was used for a short period of time.

In conclusion, EVNSRs are extremely rare tumors with only 30 cases reported to date (including this case). They occur mostly in 40-year-old patients of the female sex who are from China. EVNSRs can secrete arginine vasopressin and lead to SIAD. They are mistaken for PA. However, the presence of calcified areas, unusual in PA, and SIAD should favor the diagnosis of neurocytoma. First-line treatment is surgery. However, in subtotal resections, SIAD can be treated with empagliflozin and fluid restriction.

## Learning Points

- In the context of a patient with hyponatremia and a sellar/suprasellar tumor, hypopituitarism must be investigated and thoroughly replaced before thinking of SIAD.
- Correction of hyponatremia must be gradual to avoid osmotic stress.
- Neurocytomas are neuronal tumors that can secrete arginine vasopressin and lead to SIAD.
- SIAD is difficult to manage in the setting of a tumor-producing arginine vasopressin and, in the setting of subtotal resection, can be treated with empagliflozin and fluid restriction.
- Although these cases were diagnosed as pituitary adenomas, the unusual presentation of chronic hyponatremia in patients with a sellar and calcified lesion should favor another diagnosis.

## Data Availability

Original data generated and analyzed during this study are included in this published article.
